# Mechanism of action of microRNA166 on nitric oxide in alfalfa (*Medicago sativa* L.) under drought stress

**DOI:** 10.1186/s12864-024-10095-7

**Published:** 2024-03-28

**Authors:** Bochuang Wei, Yizhen Wang, Qian Ruan, Xiaolin Zhu, Xian Wang, Tianjie Wang, Ying Zhao, Xiaohong Wei

**Affiliations:** 1 College of Life Science and Technology, Gansu Agricultural University, Lanzhou, 730070 China; 2 Gansu Provincial Key Laboratory of Aridland Crop Science, Gansu Agricultural University, Lanzhou, 730070 China; 3 College of Agronomy, Gansu Agricultural University, Lanzhou, 730070 China

**Keywords:** MIR166, Alfalfa, Drought

## Abstract

**Background:**

Alfalfa is a perennial forage crop of high importance, but its cultivation is often affected by drought stress. Currently, the investigation of drought-related small RNAs is a popular research topic to uncover plant drought resistance mechanisms. Among these small RNAs, microRNA166 (miR166) is associated with drought in numerous plant species. Initial small RNA sequencing studies have shown that miR166 is highly responsive to exogenous nitric oxide (NO) and drought. Therefore, analyzing the expression of Msa-miR166 under nitric oxide and drought treatment is significant.

**Result:**

Bioinformatics analysis revealed that the miR166 family is widely distributed among plants, ranging from mosses to eudicots, with significant distribution differences between species. The evolutionary degree of Msa-miR166s is highly similar to that of Barrel medic (*Medicago truncatula*) and Soybean (*Glycine max*), but significantly different from the model plant Arabidopsis (*Arabidopsis thaliana*). It is suggested that there are no significant differences in miR166s within the species, and members of Msa-miR166s can form a typical stem-loop. The lowest level of exogenous nitric oxide was observed in Msa-miR166s under drought stress, followed by individual drought, and the highest level was observed after removing endogenous nitric oxide.

**Conclusion:**

In response to short-term drought, Msa-miR166s down-regulate expression in alfalfa (*Medicago sativa* L.). Exogenous nitric oxide can reduce the expression of Msa-miR166s in response to short-term drought. These findings suggest that Msa-miR166e-5p is responsive to environmental changes. The expression levels of target genes showed an opposite trend to Msa-miR166s, verifying the accuracy of Degradome sequencing in the early stage. This suggests that alfalfa experiences drought stress when regulated by exogenous nitric oxide, targeting HD ZIP-III, FRI, and CoA ligase genes. Additionally, the expression of Msa-miR166s in response to drought stress varies between leaves and roots, indicating spatiotemporal specificity.

**Supplementary Information:**

The online version contains supplementary material available at 10.1186/s12864-024-10095-7.

## Background

Alfalfa *(Medicago sativa L.)* is a valuable perennial forage crop that is rich in protein, fat, vitamins, and minerals. It contains numerous active functional ingredients and has a strong nitrogen fixation ability, resulting in high yield and economic value [1].

However, alfalfa is primarily cultivated in the arid northern regions of China where water resources are limited, particularly in the northwest. Widespread drought stress during alfalfa cultivation severely limits the yield and quality of alfalfa. In 2014, the global planting area for alfalfa was approximately 2.380 × 10^7^ hm^2^, and alfalfa grass production reached 3.41 × 10^8^ t [2]. In 2021, China produced 0.29 million tonnes of alfalfa and imported 0.52 million tonnes of alfalfa hay, according to the latest statistics from China’s State Forestry Administration. This is an urgent issue that requires serious attention. Studying the drought resistance mechanism of alfalfa and improving its drought resistance through biotechnology is significant for agriculture and animal husbandry. “San Deli” was found to be not only a high-yielding forage variety but also nutritionally advantageous after analyzing and screening the grass yield, nutritional composition, and relative feed value of 16 local and foreign lucerne varieties [3]. Previous studies have shown that “San Deli” is the most sensitive to the “star” signal molecule nitric oxide (NO) among the three varieties of alfalfa: “San Deli”, “Golden Queen”, and “Algangjin” [4]. The application of NO from an external source can significantly improve the defense response of the alfalfa cultivar “San Deli” to drought [5].

Nitric oxide (NO) is a gas signaling molecule that plays a significant role in regulating various aspects of plant growth and development [6]. Recent studies have shown that NO also plays an important role in resisting stress, particularly drought stress [7, 8]. Exogenous NO has been observed to alleviate the damage caused by drought stress in various plant species [7–13]. This phenomenon has been observed in various plant species, such as Soybean [7, 8], Banana *(Musa nana Lour*) [9], Chard *(Beta vulgaris* L.*)* [10], Rice *(Oryza sativa)* [11]_,_ Milk thistle *(Silybum marianum* L.*)* [12], and Maize *(Zea mays)* [13]. Physiologically, exogenous NO regulated fresh and dry weights of above-ground plants [7], root dry weight [10], and plant height [9]. For example, exogenous NO has been shown to promote the generation of adventitious roots in Soybean [7] and Chard [10]. It can also reduce yellowing and necrosis of Banana [9] leaves during drought, thereby improving nutrient uptake and crop yield under drought stress. This positively regulates the plant’s defense mechanisms to cope with limited water conditions [7, 9, 10]. NO also reduces abnormally increased stomatal conductance caused by drought, thereby balancing the transpiration rates and reducing the leaf water loss rate [7]. This maintains leaf water potential, improves water use efficiency, and increases resistance to drought stress [9–13]. At the biochemical level, it was found that exogenous NO can effectively improve the activity of antioxidant enzymes under drought stress and reduce the production of oxidative products [14–16]. As stress levels increase, NO can stimulate the production of secondary metabolites, including total phenolics and tocopherols [17–19]. This activation of the antioxidant system helps to maintain the balance of free radical reactive oxygen species (ROS). This phenomenon has been observed in various plant species, such as Alfalfa [5], White clover*(Trifolium repens)* [14]^14^, Soybean [8], Turfgrass [15], Tomato*(Lycopersicon esculentum Mill)* [1616], Wheat *(Triticum aestivum* L.*)* [17], Maize [17], and Malus *(Malus pumila Mill)* [18] seedlings. This reduces the inducing effect of drought on high levels of ROS, mitigates oxidative damage, preserves the normal function of plant organelles, such as chloroplasts [8, 14, 18] and cell membranes [9, 10], and alleviates water loss [9, 19] and ion leakage [6, 9, 14, 18] caused by drought. Furthermore, it has been discovered that exogenous NO can enhance the levels of photosynthetic pigments, which regulate photosynthesis and mitigate the damage caused by excess light energy [10, 12, 19]. This can alleviate the adverse effects of drought on carbohydrate accumulation and energy metabolism in plants, thereby improving drought tolerance in Chard [10], Milk thistle [12], White clover [14], and Trifoliate orange *(Citrus trifoliata L.)* [19]. In Arabidopsis [20], Rice [11], Maize [13], and Malus seedlings [18], it has been discovered that NO causes osmotic regulation by upregulating the accumulation of pressure-compatible solutes. This reduces ion leakage caused by drought and the accumulation of malondialdehyde and soluble proteins in leaves, thereby alleviating the adverse effects of drought on plants [11, 13, 18]. At the molecular level, plants respond to drought by sensing external drought stimuli through sensors in the biofilm and transmitting information through various signal transduction pathways [5, 14, 20]. This results in alterations to the expression and adaptation of genes that respond to drought [5, 14, 20]. These genes are regulated by complex mechanisms, including transcriptional cascade reactions [6]. Plant miRNAs are a type of endogenous small, single-stranded non-coding RNA molecules. They are approximately 20–24 nt in length and are derived from longer precursor (pre-miRNA) molecules with stem-loop structures [21]. In plants, mature miRNAs target mRNA after transcription through gene silencing complexes, resulting in the transcriptional silencing and regulation of target genes [22]. Plants respond to stress by stimulating signal transduction substances, stress-protective substances, and secondary metabolites [23, 24].

MiR166 plays a critical role in several developmental processes and regulates biotic and abiotic stresses in major crops [25]. It is particularly effective in response to abiotic stresses such as drought [27], salinity [28, 29], temperature [31], and excess cadmium [24]. The morphological development of plants is altered, enhancing their stress resistance [26]. The miR166 plays a critical role in the response to drought stress in plants such as Maize [24], Rice [27] and Arabidopsis [26]. A silencing vector for miR166 was constructed in the dicotyledonous plant Arabidopsis [26] using short tandem target mimic technology (STTM) to obtain the maize silencing the miR166 strain: STTM166. It was found that Rice-STTM166 [27] and Maize-STTM166 [28] showed significantly greater drought resistance than the wild-type. Phylogenetic analysis and Reverse-Transcription Quantitative PCR (qRT-PCR) revealed that drought significantly affects the expression of MiR166s and their target genes in plants, including Highbush Blueberry [29], Soybean [30], Tea [31], and *Dimocarpus longan* Lour [32]30. Under drought treatment, the expression of MiR166 decreased to varying degrees in Soybean [30] and Highbush Blueberries [29], with some showing downregulation followed by upregulation. This particular miRNA targets primarily the HD-ZIP III gene family, which comprises a significant number of members [25]. Several studies have demonstrated that HD ZIP-III plays a crucial regulatory role in the development of plant vascular tissue [33]. This improves drought resistance by modifying plant vascular development and leaf structure [34, 35]. The study used qRT-PCR to identify 42 Zmhdz9 genes with varying expression patterns during drought and rehydration treatment in maize [36]. Thirteen genes were upregulated, three genes were downregulated, and 26 genes exhibited an irregular expression trend [37]. Furthermore, overexpression of the alfalfa Mfhb-1 gene significantly improved drought tolerance in both Arabidopsis and alfalfa [37]. The overexpression of ATHB-7 in Arabidopsis and Tobacco significantly enhanced their drought resistance [38]. It was observed that ATHB-7 was mainly expressed in the root system during the seedling stage in Arabidopsis. The overexpression of ATHB-6 has been found to enhance drought resistance in maize and promote root growth [39].

Alfalfa is a leguminous plant genus that is renowned for its high protein and vitamin content, earning it the nickname “king of forage”. However, alfalfa is typically susceptible to drought stress during the growing season. Therefore, it is essential to conduct further research into alfalfa’s response to stress and the discovery of small RNAs and genes related to stress resistance to promote the breeding and improvement of alfalfa. Recent advances in high-throughput sequencing technology and bioinformatics have enabled the identification and analysis of the alfalfa miR166 family at the whole-genome level. While the function of miR166 has been reported in other plants, its role in alfalfa has been scarcely investigated. This study screened Msa-miR166s from previous data [40] and systematically analyzed the evolutionary analysis, stem ring structure, and expression profile of members of this family. The expression differences of MiR166s and its target genes in roots and leaves under drought and nitric oxide were analyzed using qRT-PCR. The spatiotemporal expression of MiR166s and their target genes in alfalfa provides a foundation for further research on the function of MiR166s in alfalfa.

## Materials and methods

### Plants’ material

The selected seeds of the alfalfa variety “San Deli” were provided by Jiuquan Future Grass Industry Co., LTD. The scientific name of Suntory is *Medicago sativa* L. The variety was registered under No. 247 on December 11, 2002. The applicants were Chen Gu et al. and Bailu Tianjin International Co., LTD. The material for the qRT-PCR experiments in this article, alfalfa (“San Deli” variety *Medicago sativa* L.), was grown indoors in 2023. Each pot was sown with approximately 20 seeds. All seedlings were grown at a temperature of 25 °C under the same photoperiod of 14 hours of light and 10 hours of darkness. They were watered every 2 days and regularly supplied with Hoagland nutrient solution. The treatment began when the “San Deli” seedlings were in the seedling stage, 45 days after growth. Each experimental and control group consisted of eight pots of 45-day-old alfalfa plants. The study involved two experimental groups and a blank control group. The first experimental group was administered exogenous nitric oxide through a 0.1 mmol/L Sodium Nitroprusside (SNP) solution, while the second experimental group was administered 200 μM Carboxy-PTIO potassium salt (PTIO) solution to clear endogenous nitric oxide. During the pretreatment, the leaves were sprayed once every evening for four consecutive days until the end of the drought treatment. As a control variable, the control group was sprayed with ddH_2_O during the pretreatment, similar to the experimental group. On the fourth day, after 3 days of continuous pretreatment, all experimental materials were subjected to drought stress by irrigating their roots with a 15% PEG-6000 solution. Samples were collected at 0, 6, 12, and 24 hours after irrigation. The control group in question was named the PEG group. The samples were rinsed with ddH_2_O, dried, randomly collected, and stored at − 80 °C. Small RNA Sequencing (Small RNA-seq) [40] and degradome sequencing experiments in 2019 and 2020, respectively. The experimental materials were divided into three groups: a drought-treated group, a drought-treated group after NO pretreatment, and a blank control group. All other treatment conditions were the same as previously described.

### Acquisition of Msa-miR166 family member

The miR166 family members and their target sequences of alfalfa were obtained from our experimental group’s previous Small RNA-seq and Degradome Sequencing. We obtained four pre-miR166s, namely Msa-miR166a, c, d, and e, along with four mature sequences, namely Msa-miR166a, c, e-5p, and g-5p, through Small RNA-seq in 2019. The precursor sequences are shown in Supplementary Table 1. The Degradome sequencing of the “Xinjiang Daye” variety of alfalfa [41] revealed six new mature Msa-miR166s. Upon comparison, they were identified as Msa-miR166b, d, f, g-3p, and e-3p, with only two sequences differing from the known Msa-miR166s sequence. Consequently, the primers designed for these six different Msa-miR166s were used for the qRT-PCR experiment.

The target gene of Msa-miR166s was preliminarily predicted through degradome sequencing with reference to alfalfa genome data. Supplementary Table 3 shows the corresponding target gene sequence of Msa-miR166. Primers for the qRT-PCR experiment were designed based on six selected genes.

### Statistical analysis of MiR166 distribution in plants

This article uses a total of 293 pre-miR166 sequences and 358 mature sequences from 50 species, including 289 pre-miR166s and 350 mature miR166s downloaded from the miRbase website (http://www.miRbase.org/) and data from alfalfa obtained from preliminary experiments. Using the botanical classification from the miRbase website and the phylogenetic tree of the APG IV system (https://duocet.ibiodiversity.net/index.php?title=APG_IV%E7%B3%BB%E7%BB%9F), we conducted a quantitative statistical analysis to investigate the distribution of tMiR166s across various families, genera, and species.

### Multiple sequence alignment and phylogenetic analysis of Msa-miR166s

The mature sequence of Msa-miR166s was compared using Dnaman 8.0 software. A Web Logo was generated using online software (https://weblogo.berkeley.edu/logo.cgi) to illustrate the degree of motif conservation in the mature body conservative sequence. The Neighbor-joining (NJ) method and bootstrap of MEGA11 software [41, 42] were used to perform 1000 bootstrap tests. The phylogenetic tree for pre-miR166s and the mature miR166 sequence of several species were constructed. The related members of alfalfa were then identified.

### Prediction of the secondary structure of MIR166s and their target genes

The mfold web server (http://unafold.rna.albany.edu/?q=mfold) was used to predict the secondary structure of Msa-miR166 precursor transcripts. Target genes for Msa-miR166s were predicted by analyzing Degradome sequencing data.

### RNA extraction, reverse transcription and qRT-PCR

Following sample collection, total RNA was extracted using the UNIQ-10 column Trizol total RNA extraction kit (Sangon Bioengineering Co., Ltd., Shanghai) and the TIAN Script II RT Kit (Tiangen Biochemical Technology Co., Ltd., Beijing). RNA concentration and purity were examined using Nanodrop 2000 (Thermo Fisher Scientific, USA), RNA integrity was examined through agarose gel electrophoresis, and RIN values were determined by Agilent 5300 Bioanalyzer (Agilent, USA).

The TaqMan™ Advanced miRNA cDNA Synthesis Kit (Tailing Reaction, ThermoScientific) was used to synthesize miRNA cDNA templates for qRT-PCR. The qRT-PCR analysis employed specific forward primers and universal reverse primers designed for mature miR166. The primer sequence can be found in Supplementary Table 2. The U6 gene was used as the internal reference gene for Msa-miR166s. The selection of internal reference genes was based on the results of Yao-dong Zhao [40].

The cDNA was transcribed using the Evo M-MLV Reverse Transcription Premixed Reagent Kit (which includes a gDNA removal reagent for qRT-PCR) Ver.2 (Accurate Biology). Primers for the target genes were designed using NCBI Premier-BLAST (see Supplementary Table 4). The amplified fragments were approximately 100–200 bp. The elongation factor 1 alpha, EF-1α, was used as the internal reference gene for the target gene in the root, while the 18SrRNA was used as the internal reference gene for the target gene in the leaves. The internal reference gene sequences are presented in Supplementary Table 4. The selection of these genes was primarily based on Fu Yuanyuan’s results [43]. The entire experiment was conducted using the Quantageneq225 qRT-PCR instrument, and the primer sequences were synthesized by Shenggong Biotechnology (Shanghai) Co., Ltd.

For statistical analysis and data calculation, Microsoft Excel (Office 2021) was utilized. The analysis of variance was conducted using SPSS Statistics 19.0 software (SPSS Inc., Chicago, IL), and the mean values were compared using a student’s t-test at a 5% significance level. The results represent the average of three repeated experiments. The relative expression of miRNA and predicted target genes under different treatment times was calculated using 2^-ΔΔCT^, and the results are displayed as log_2_FC. This method was described in [44].

## Results

### Statistical analysis of MiR166 distribution in plants

Table 1 shows the distribution of the miR166 family across dicotyledons, monocotyledons, and other angiosperms, as well as gymnosperms such as China fir (*Cunninghamia lanceolata*, *Picea abies*), P. densata (*Pinus densata*), and loblolly pine (*Pinus taeda*), and older ferns such as Herba Selaginellae Moellendorfii (*Selaginella moellendorfii*) and *Physcomitrella patens* (*Bryophyte Physcomitrella patens*). The number of miR166 family members varies significantly among different species in terms of quantity. For instance, the number of mature MiR166 bodies in Leguminosae varies from 1 in Birdsfoot trefoil (*Lotus corniculatus)* and Bean (*phaseolus vulgaris*), to 27 in Soybean, and 10 in Barrel Medic. In the Gramineae family, Maize has 26, Rice has 24, Purple Falsebrome (*Brachypodium distachyon*) has 18, Sorghum (*Sorghum bicolor*) has 11, Goat Grass (*Aegilops tauschii*) has 10, Barley (*Hordeum vulgare*) has 3, Sugarcane (*Saccharum SP*) has 1, and Tall Fescue (*Festuca arundinaceous*) has 1 (refer to Table 1). The distribution of miR166s is uneven within certain families and genera. For example, within gymnosperms, China fir has the lowest number of mature sequences, with only one. Similarly, monocotyledonous grasses in angiosperms, such as Tall Fescue and Sugarcane, have only one mature sequence. There is only one complete sequence for dicotyledonous angiosperms, such as the legumes Birdsfoot Trefoil and *Phaseolus vulgaris*, the trifoliate orange of the Rutaceae, and *Gossypium hirsutum* of the Malvaceae. Additionally, there are species with only one mature sequence in Compositae. The Leguminosae Soybean had the highest number of MiR166s, with a total of 26. Maize had the second-highest number, also with a total of 26.

**Table 1:** Quantitative statistics of MicroRNA166 family members in different plants

Type				Number of pre-miRNA/mature miRNA
Division		Family	Species(Abbreviation)	
bryophyta		Funariaceae	*Physcomitrella patens(ppt)*	13/13
fern		Selaginellaceae	*Selaginella moellendorffii(smo)*	3/3
gymnosperm		Cupressaceae	*Cunninghamia lanceolata(cln)*	1/1
		Pinaceae	*Picea abies(pab)*	10/10
			*Pinus densata(pde)*	2/2
			*Pinus taeda(pta)*	3/3
angiosperm	Base group	Amborellaceae	*Amborella trichopoda(atr)*	4/4
	Monocotyledons	Asparagales	*Asparagus officinalis(aof)*	4/4
		Bromeliaceae	*Vriesea carinata(vca)*	3/6
		Poaceae	*Brachypodium distachyon(bdi)*	10/18
			*Aegilops tauschii(ata)*	5/10
			*Festuca arundinaceous(far)*	1/1
			*Sorghum bicolor(sbi)*	11/11
			*Zea mays(zma)*	14/26
			*Saccharum sp(ssp)*	1/1
			*Oryza sativa(osa)*	14/24
			*Hordeum vulgare(hvu)*	3/3
	Dicotyledoneae	Ranunculaceae	*Aquilegia caerulea(aqc)*	5/5
		Vitaceae	*Vitis vinifera (vvi)*	8/8
		Leguminosae	*Glycine max(gma)*	21/26
			*Lotus japonicus(lja)*	1/1
			*Medicago truncatula(mtr)*	8/10
			*Phaseolus vulgaris(pvu)*	1/1
		Rosaceae	*Fragaria vesca(fve)*	6/7
			*Malus domestica(mdm)*	10/10
			*Prunus persica(ppe)*	5/5
		Cucurbitaceae	*Cucumis melo(cme)*	9/9
		Salicaceae	*Populus trichocarpa(ptc)*	17/17
		euphorbiaceae	*Ricinus communis(rco)*	5/5
		Linaceae	*Linum usitatissimum(lus)*	11/11
		Myrtaceae	*Eugenia uniflora(eun)*	1/2
		Rutaceae	*Citrus reticulata(crt)*	2/2
			*Citrus sinensis(csi)*	13/24
			*Citrus trifoliata(ctr)*	1/1
		Malvaceae	*Gossypium hirsutum(ghr)*	1/1
			*Gossypium raimondii(gra)*	2/2
			*Theobroma cacao(tcc)*	4/4
		Caricaceae	*Carica papaya(cpa)*	5/5
		Cruciferae	*Arabidopsis lyrate(aly)*	8/16
			*Arabidopsis thaliana(ath)*	7/10
			*Brassica napus(bna)*	6/9
			*Camelina sativa(cas)*	6/8
		Solanaceae	*Nicotiana tabacum(nta)*	8/8
			*Solanum lycopersicum(sly)*	3/4
			*Solanum tuberosum(stu)*	4/7
		Plantaginaceae	*Digitalis purpurea(dpr)*	2/2
		Labiatae	*Salvia sclarea(ssl)*	2/2
		Asteraceae	*Helianthus paradoxus(hpa)*	1/1
			*Helianthus petiolaris(hpe)*	1/1

Before the emergence of monocotyledons, the number of mature bodies in mir166 was equal to the number of precursors. However, in monocotyledons, the number of mir166 mature bodies exceeds the number of precursors. In 16 species of angiosperms, the number of mature bodies exceeded the number of precursors, while in 25 species, the number of mature bodies was equal to the number of precursors. Alfalfa belongs to the dicotyledonous plant family Leguminosae, and together with Barrel Medic are the two most important species in the genus Alfalfa. Barrel Medic has 8 miR166 precursors and 10 mature bodies; Soybean, which is also a legume, has 21 miR166 precursors and 26 mature bodies.

### The evolutionary analysis of the pre-miR166 family

**Fig. 1:**
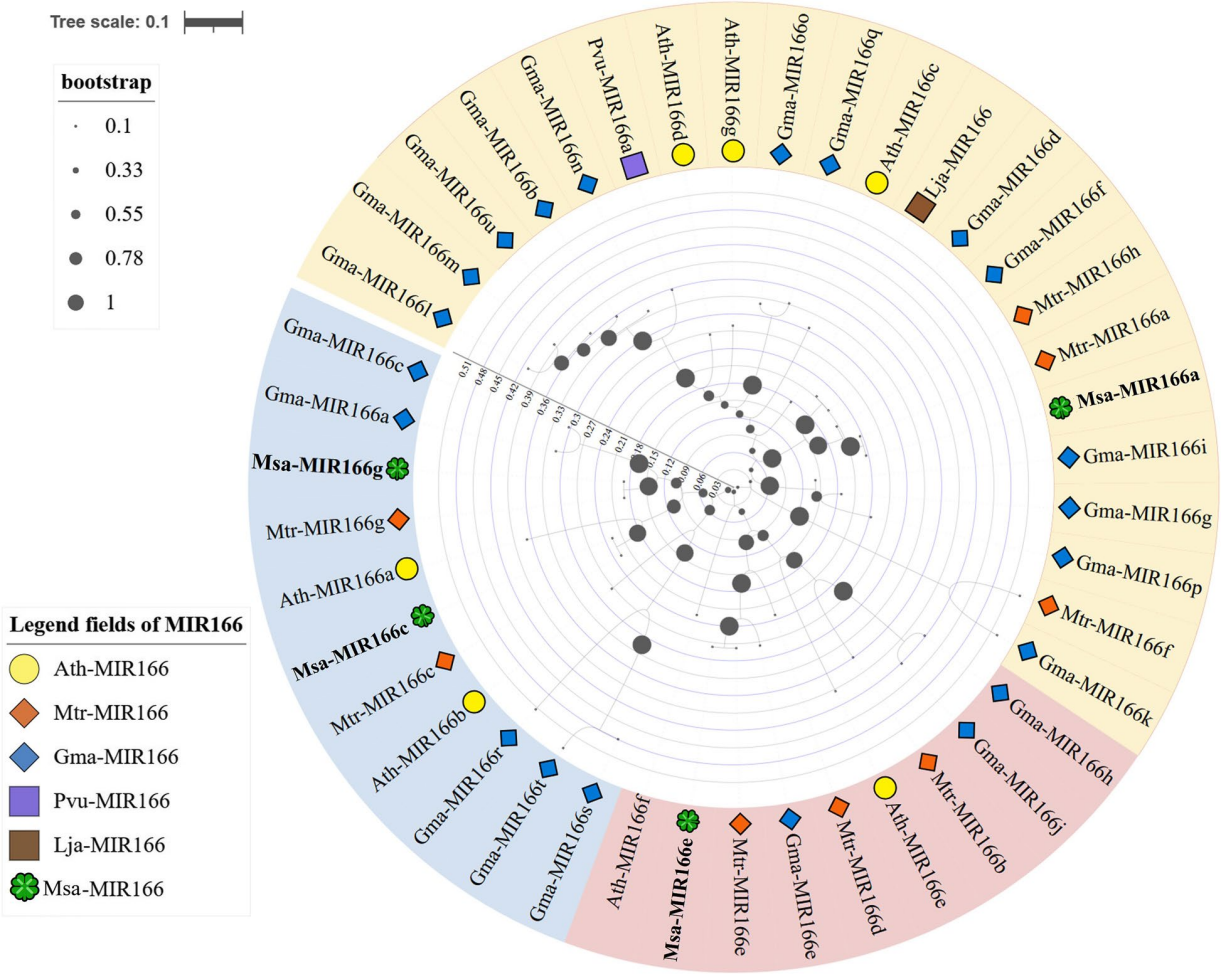
Evolutionary tree of pre-miR166 in leguminous plants and Arabidopsis model plant The scale represents the average number of amino acids at each site

The pre-miR166s of Arabidopsis, Soybean, *Tribulus Terrestris*, Birdsfoot trefoil, and *Phaseolus vulgaris* of Leguminosae were obtained from the miRBase database. Arabidopsis has 7 members, Soybean has 21 members, *Tribulus Terrestris* has 7 members, Birdsfoot trefoil has 1 member, and *Phaseolus vulgaris* has 1 member. The Small RNA-seq data obtained four pre-miR166s in Alfalfa. Figure 1 shows the phylogenetic tree of the miR166 family, which is divided into three branches. The Pick branch includes miR166f and miR166d of Arabidopsis, miR166e and miR166b of Barrel Medic, and miR166j and miR166h of Soybean. The blue branch comprises miR166a and b of Arabidopsis, miR166a, c, r, t, and s of soybean, miR166b, c, and g of barrel medic, and miR166c and g of alfalfa. The yellow branch includes additional members of MiR166s discovered in Soybean, Arabidopsis, Barrel Medic, and Alfalfa, as well as all members of Birdsfoot trefoil and *Phaseolus vulgaris*. The evolutionary tree shows that Msa-miR166a is most closely related to Mtr-miR166a, followed by Mtr-miR166h. Msa-mir166c is most closely related to Mtr-miR166c, followed by Ath-miR166a. Msa-mir166e is most closely related to Mtr-miR166e, followed by Gma-miR166e. Finally, Msa-miR166g is most closely related to Mtr-miR166g, followed by Gma-miR166a and Gma-miR166c. Msa-miR166e is the longest branch on the evolutionary tree among the four known precursors of alfalfa. Its branch is of equal length to the evolutionary branches of Ath-miR166b, d, and g.

**Fig. 2:**
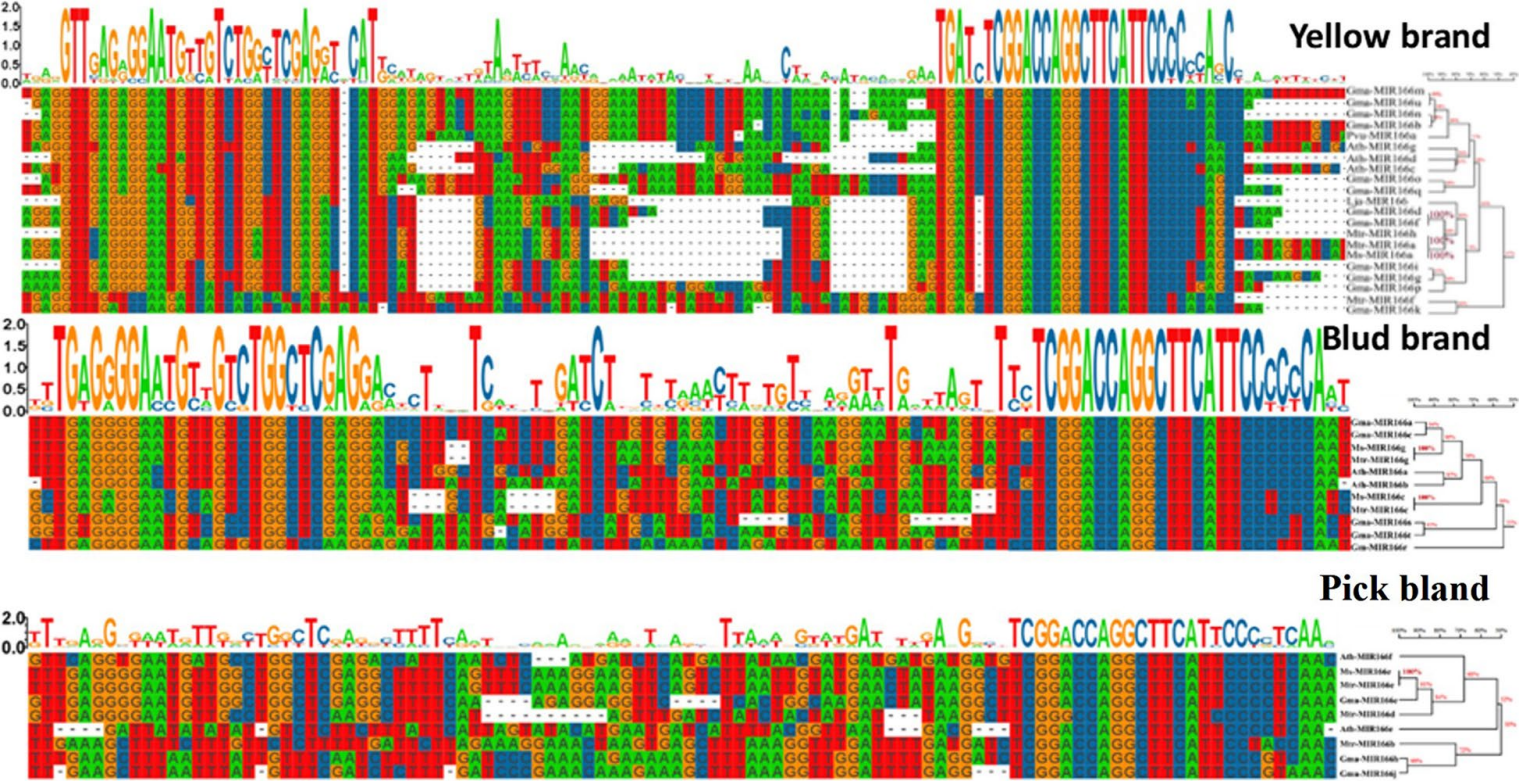
Conservative motifs in the branch of the pre-miR166 evolutionary tree The yellow brand corresponds to the branch with a base colour of yellow in the evolutionary tree of Fig. 1; the Blud brand corresponds to the branch with a base colour of blue in the evolutionary tree of Fig. 1; and the Pick bland corresponds to the branch with a base colour of pink in the evolutionary tree of Fig. 1

The precursor sequences of Msa-miR166 members were analyzed using DNAMAN and MEAG11 alignment tools across various branches (refer to Fig. 2). The analysis revealed that precursor sequences with the same length had a high similarity of 100%, with a minimum of 89%. Figure 2 shows that Msa-miR166s are highly conserved. Create a conservative motif diagram in TBtools with base sequence directions ranging from 5′ to 3′. The conservative motifs of each branch exhibit significant differences, but a highly conservative motif is present at the 3′ end. In contrast, the conserved pattern of miR-166 at the 5’ end is unstable. In particular, the part of the pink branch where Msa-miR166e is located.

### Prediction of the secondary structure of MIR166s and their target genes

**Fig. 3:**
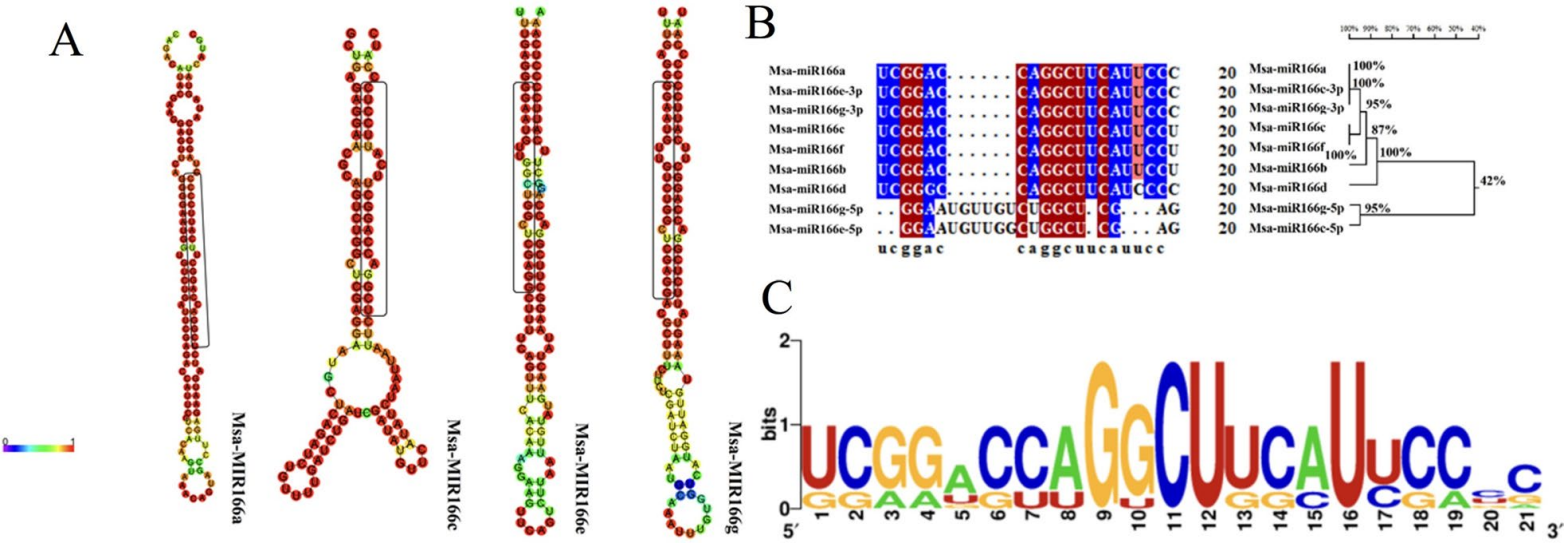
Conserved bases and conserved motifs map of predicted secondary structure of Msa-miR166 family precursors with mature body sequences, **A** Stem-loop Structure of Msa-miR166 Precursor Family Members, the mature miRNA portion is highlighted in a black box; **B** Base Conservation Analysis of Mature Body Sequences of Msa-miR166 Family; **C** Conservative motif diagram of mature body sequences of Msa-miR166 family

The secondary structure of Msa-miR166 precursor transcripts was predicted using the mfold web server, while the target genes of Msa-miR166s were predicted using degradome sequencing analysis. The secondary stem-loop structure of the precursor sequences of the Msa-miR166 family members is predicted online according to the system defaults of the RNA folding form built into the mfold web server website. The miR166 family has typical RNA hairpin structures that correspond to the basic structural characteristics of miRNA. Among them, the free energy of Msa-miR166a is − 48.6 kcal/mol, the free energy of Msa-miR166c is − 42.80 kcal/mol, the free energy of Msa-miR166e is − 50.2 kcal/mol, the free energy of Msa-miR166g is − 45.7 kcal/mol, and the average free energy is − 46.5 kcal/mol. The free energy of Msa-miR166a and Msa-miR166e is higher than the average free energy. There are differences in the length of the precursor sequences among the four members of the Msa-miR166 family, with Msa-miR166a being the shortest and Msa-miR166g being the longest. Secondary structure analysis of the precursor sequences showed that the precursor sequences of all four Msa-miR166 members could form stable stem-loop structures. The mature miRNA sequences of Msa-miR166a and Msa-miR166c were located at the 3′ end, while those of Msa-miR166e and Msa-miR166g were located at the 5’ end (Fig. 3A).

The target genes, Transcript ID, and splicing sites of Msa-miR166a, b, c, d, e-5p, and g-5p were predicted by referring to the Alfalfa genome of “Xinjiang Daye” varieties. Through nonparametric analysis of Degradome Sequencing (see Fig. S1), it was observed that Msa-miR166a, b, and c regulate the expression of target gene *Ms-ATHB-15*, *Ms-ATHB-8*, and *Ms-REV* through the same cleavage site. Similarly, Msa-miR166b and Msa-miR166c regulate the expression of the target gene *Ms-CoA ligase* through the same cleavage site. In addition, Msa-miR166e-5p and Msa-miR166g-5p respectively regulate the expression of target genes *Ms-FRI* and *Ms-SF1* (Table 2).

**Table 2:** Target genes and their cleavage sites corresponding to Msa-miR166s

Msa-miR166s	Target Gene	Transcript ID	TSlice	DegradomeCategory	DegradomePval
Msa-miR166a	*ATHB-15*	MS.gene069869	553	0	0.00045
	*ATHB-8*	MS.gene40724	556	0	0.00229
	*REV*	MS.gene08703	580	0	0.00504
Msa-miR166b	*CoA ligase*	MS.gene003303	1220	0	0.00549
	*ATHB-15*	MS.gene069869	553	0	0.00137
	*ATHB-8*	MS.gene40724	556	0	0.00229
	*REV*	MS.gene08703	580	0	0.00526
Msa-miR166c	*CoA ligase*	MS.gene003303	1220	0	0.00549
	*ATHB-15*	MS.gene069869	553	0	0.00137
	*ATHB-8*	MS.gene40724	556	0	0.00229
	*REV*	MS.gene08703	580	0	0.00526
Msa-miR166d	*ATHB-8*	MS.gene40724	556	0	0.00045
	*REV*	MS.gene08703	580	0	0.00343
Msa-miR166e-5p	*FRI*	MsG0880042046	1704	2	0.37533
Msa-miR166g-5p	*SF1*	MsG0480019689	1419	2	0.04909

TSlice is the binding site of miRNAs to the mRNA of the target gene, the 10th nucleotide at the 5’ end of the degradation fragment paired with miRNA; Degradome Category 0: The original data fragment is at this position, with an abundance equal to the maximum abundance on the transcribed RNA, and only one maximum value; Category 1: The abundance of the original data segment at this position is equal to the maximum abundance value on the transcribed RNA, and there is more than one maximum abundance value; Degradome Category 2: The abundance of the original data segment at this location is less than the maximum abundance on the transcribed RNA, but greater than the median abundance on the transcribed RNA

By analyzing the primary and secondary structures of the target genes (Supplementary Table 5), it was found that the target genes regulated by the Msa-miR166s are mostly hydrophobic proteins and that the structures of *Ms-ATHB-8* and *Ms-REV*, both belonging to the HD ZIP III family, are similar.

## The evolutionary analysis of the mature miR166s of plants

### Mature miR166 characteristics in alfalfa

Comparative searches in the mirBase database, using Msa-miR166 sequences, revealed that Msa-miR166s matched extremely well with miR166s from Barrel Medic, Soybean, Maize, and Rice (Table 3). Both known and newly discovered miR166s in alfalfa can be found in other species with known mature miR166s with the same base sequence. It can be seen that Msa-miR166s is very conservative in the evolution of plant miR166s (Table 3).

**Table 3:** Characteristics of eight mature miR166s in Alfalfa “San Deli” varieties

ID(Msa- miR166-)	Length (nt)	Homologs	Species	Score/Evalue
a	21	Gma-166i, c-3p, d, e, f, G; Bdi-miR166b, c, d -3p; et al.	Soybean, *Brachypodium distachyon*, et al.	105/0.002
c	21	Mtr-miR166c, f; Osa-miR166g, h-3p; ZmamiR166l, m-3p; et al.	Barrel medic, rice, Maize, et al.	105/0.002
e-5p	22	Mtr-miR166e-5p, Osa-miR166h-5p, Zma-miR166m-5p.	Barrel medic, Rice, Maize	105/0.002
g-5p	21	Mtr-miR166g-5p, gma-miR166a, c, d-5p, I; et al.	Barrel medic, Soybean, et al.	105/0.002
d	21	Mtr-miR166d	Barrel medic	105/0.002
b	21	Mtr-miR166b	Barrel medic	105/0.002
f	Same sequence as Msa-miR166c			
g-3p	Same sequence as Msa-miR166a			
e-3p	Same sequence as Msa-miR166a			

NMa is All Zero, NMa: Differential base number between Msa-miR166s and their homologous miRNA

### Sequence alignment of mature Msa-miR166s

The Msa-miR166 family members’ internal evolution tree was created using DNAMAN 8.0 software. The results were classified into two categories: Msa-miR166g-5p and Msa-miR166e-5p were grouped, while the remaining members were grouped separately. The similarity between Msa-pre-miR166a and Msa-pre-miR166e-3p sequences was high, reaching 95.79%. Upon analyzing eight mature sequences of Msa-miR166 family members, it was discovered that the mature sequences of Msa-miR166 members with the same length were entirely consistent (Fig. 3B).

Web Logo was used to generate the logo of the conserved sequence of the mature body of the miR166 family (Fig. 3C). The conservative motif is visible, including a G base at the ninth position, a C base at the eleventh position, and U bases at the twelfth and sixteenth positions.

### Evolutionary analysis of mature miR166s in plants

**Fig. 4:**
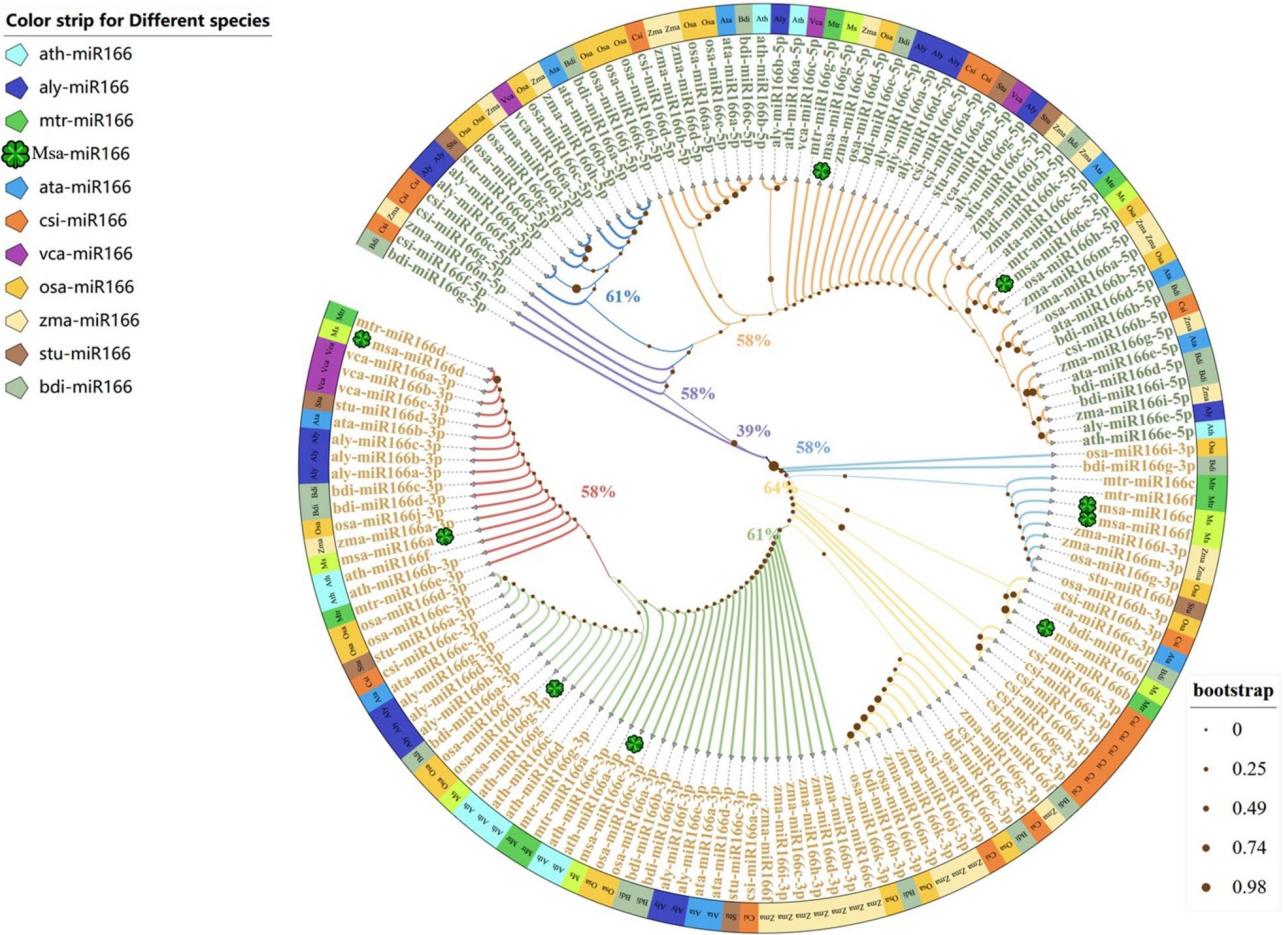
Evolutionary tree of 154 mature MiR166s from 11 species in 6 families and genera Note: The color of the outer ring color bar represents the colors of different species; The label color represents the grouping of the miR166–3 and -5 terminals; Different branch colors represent the detailed grouping of -3p and -5p, and the percentage at the branch represents the credibility of its grouping; Alfalfa species are labeled  as an identifier

The evolutionary tree shows that the -3p and − 5p of MiR166 differentiate into two branches, when there is only one mature sequence, most of it is located in the -3p branch (Fig. 4). All seven mature sequences of Msa-miR166a, d, b, c, f, g-3p, and e-3p are located on the -3p branch of the evolutionary tree, except for Msa-miR166g-5p and e-5p on the -5p branch(Fig. 4). The evolutionary tree has no obvious root node, its branches are sequential, and the eight alfalfa miR166s are sequentially distributed on the different branches of the tree. In addition, the phylogenetic tree shows that Msa-miR166s have the closest relationship with Leguminous dicotyledonous plants such as Barrel medic and Soybean, followed by Gramineae of Monocotyledon such as Maize and Rice, Poaceae (Fig. 4).

## Analysis of the expression patterns of Msa-miR166s and its target genes in young alfalfa seedlings

### Total RNA isolation related gel images

**Fig. 5:**
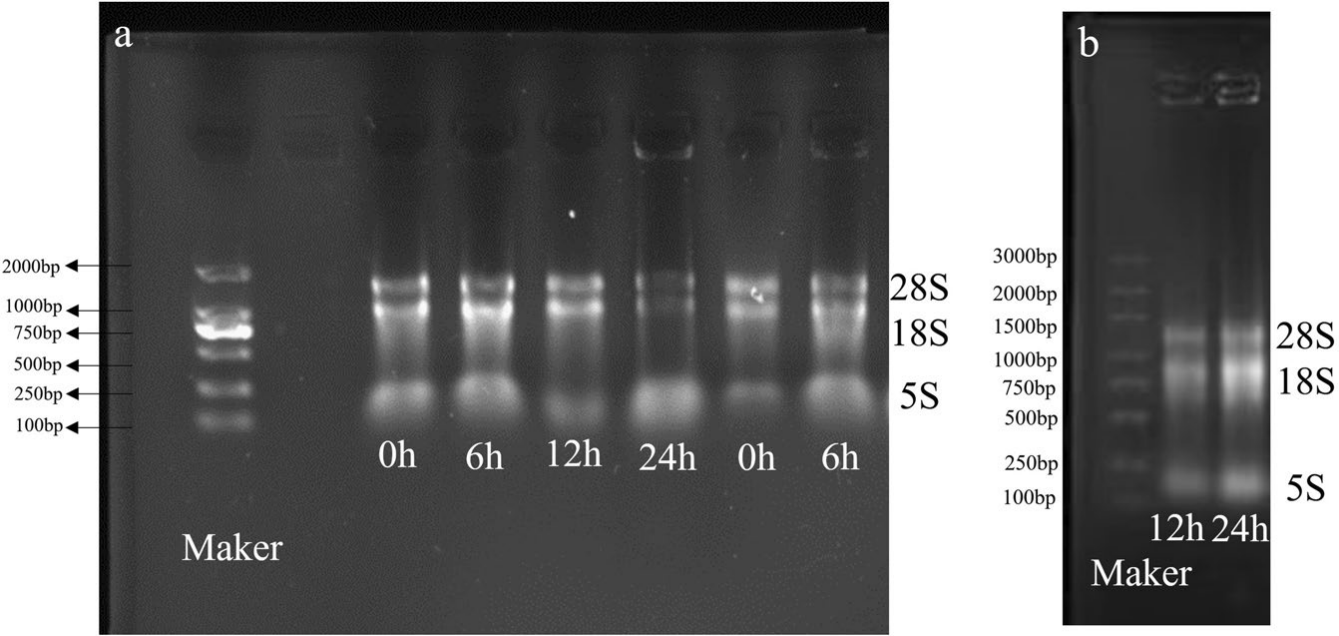
Total RNA isolation related gel images From left to right, samples were taken at 0 h, 6 h, 12 h, and 24 h in both roots and leaves

RNA was extracted from 12 samples and subjected to agarose gel electrophoresis. The resulting gel showed three distinct bands: 28S, 18S, and 5S (Fig. 5). These bands can be used as qRT-PCR experimental templates for the next step.

### Results of qRT-PCR

**Fig. 6:**
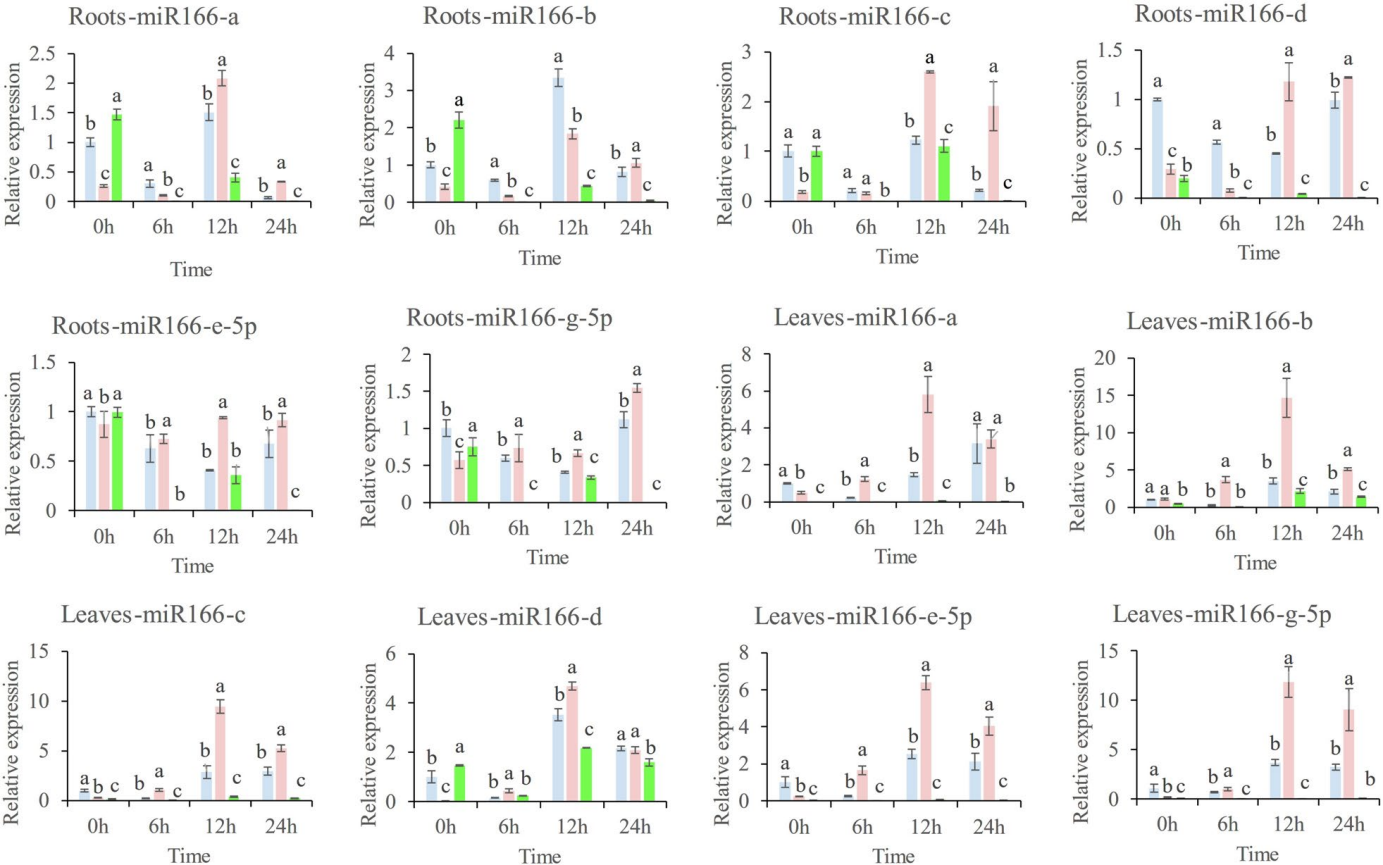
Relative expression of Msa-miR166s in Roots and leaves Note: 0 h: The experiment involved alfalfa plants that were not subjected to drought treatment, as well as those that were treated with drought for 6, 12, and 24 hours. Each group, both experimental and control, consisted of three pots of plants. The PEG group is represented by blue bars, the PTIO group by pink bars, and the SNP group by green bars. Error bars indicate the standard error (SE) of three biological and three technical replicates. Different letters indicate significant differences (*p* < 0.05) between samples, as determined by Tukey’s test. The values were normalized using the U6 gene

As illustrated in Fig. 6, the expression level of Msa-miR166s in the root remained constant in the PEG group at 0 hours. During the progression of the drought, the expression of mir166a, b, c decreases within 6 hours after the onset of drought, but rapidly increases at 12 hours, surpassing the expression level at 0 hours. The minimum increase is 1.2 times and the maximum increase is 3 times. However, at 24 hours, it rapidly decreased once more. After the drought, the expression levels of Msa-miR166d, e-5p, and g-5p gradually decreased and then increased, reaching their lowest value at 12 h, which was 0.4 times that of the 0 h. At 24 hours, the expression level increased once more and remained consistent with the level observed at 0 hours. Following the removal of NO from the plant body through PTIO treatment, the expression level of miR166s decreased at 0 h. However, at 6 h of drought, the expression level of miR166s did not change significantly. Even Msa-miR166a, b, d, e-5p showed a downward trend. However, at 12:00, the expression level of Msa-miR166a, b, c, and d increased to its maximum, which was approximately five times that of PTIO at 0 h. There was no significant change in the expression level of Msa-miR166e-5p or g-5p after 12 hours of drought. After 24 hours, the expression levels of Msa-miR166a, b, and c decreased, while the expression level of Msa-miR166d and Msa-miR166e-5p remained almost unchanged. Additionally, the expression level of Msa-miR166g-5p increased by almost double. After applying external NO, the expression level of Msa-miR166s in roots fluctuated at 0 h. However, after drought treatment, the expression level of mir166s rapidly decreased by three orders of magnitude. At 12 h after drought, there was a slight rebound, still significantly lower than the expression level at 0 h; At 24 h, it rapidly decreased to the level at 6 h.

Under drought conditions, the expression changes of Msa-miR166s in leaves were consistent with those in roots. After the drought, all values decreased before increasing again. However, the peak reached was larger than that of the roots. Apart from Msa-miR166b, the expression levels of the other Msa-miR166s changed after 12 and 24 hours of drought. For instance, Msa-miR166a reached its maximum value in the leaves after 24 hours, which was later than the peak observed in the roots. In the PTIO group, the expression levels of Msa-miR166s were consistent between leaves and roots at 0 h and 6 h under continuous drought. However, at 12 h, the expression levels in leaves reached their maximum values and decreased slightly at 24 h, remaining slightly lower than the expression levels at 12 h. In the group of SNPs, the Msa-miR166s present in the leaves were distinct from those found in the roots. The expression level was lower before the drought and decreased again at 6 hours of drought, but increased at 12 hours. The peak value remained lower than that of the PEG group at the same time and decreased again after 24 hours. The expression level of Msa-miR166s in the PEG group was 100 times higher than that in the SNP group.

**Fig. 7:**
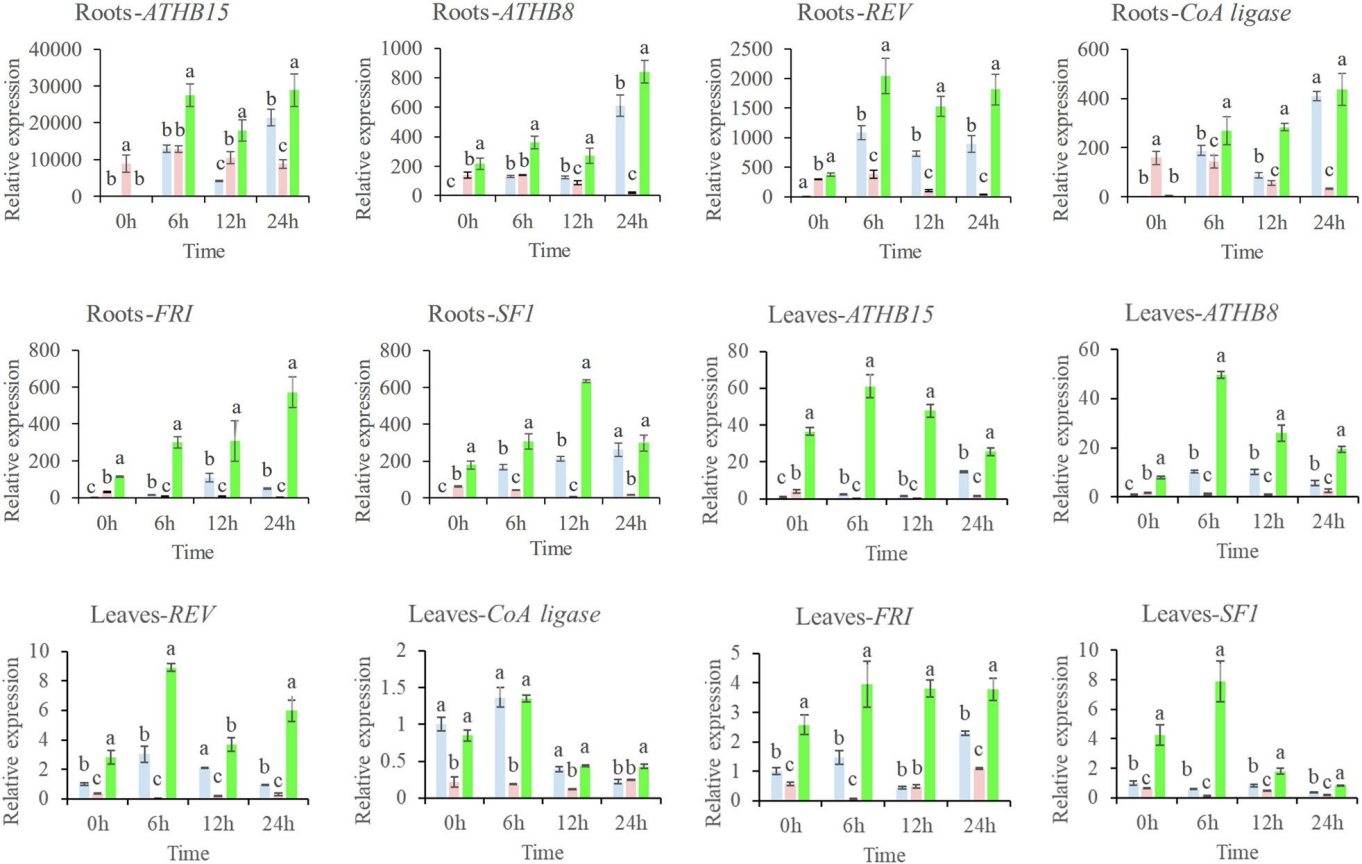
Expression of the target gene in roots and leaves Note: 0 h: The experiment involved alfalfa plants that were not subjected to drought treatment, as well as those that were treated with drought for 6, 12, and 24 hours. Each group, both experimental and control, consisted of three pots of plants. The PEG group is represented by blue bars, the PTIO group by pink bars, and the SNP group by green bars. Error bars indicate the standard error (SE) of three biological and three technical replicates. Different letters indicate significant differences (*p* < 0.05) between samples, as determined by Tukey’s test. The values were normalized against the 18SrRNA gene in the leaves and EF-1α in the roots

In comparison to the expression changes of Msa-miR166s, the expression changes of these six genes exhibit an opposite trend (refer to Fig. 7). This confirms the results of degradation group sequencing and preliminarily identifies these six genes as the target genes of Msa-miR166s. After the drought treatment, the target genes of the SNP group in the roots gradually upregulated, and slightly flattened or decreased at 12 h. This is consistent with the slightly increased expression of Msa-miR166s in the roots at 12 h. The target genes in leaves that are affected by the SNP group exhibit a baseline expression at 0 h. Following drought treatment, their expression levels peak at 6 h and subsequently decrease to varying degrees. The expression of Msa-miR166 in leaves showed consistency with the fluctuation observed during drought, with low levels at 0 h and 6 h and high levels at 12 h and 24 h.

## Discussion

The alfalfa “San Deli” variety stands out among forage plants for its high yield and quality, but its cultivation is usually limited by drought [2, 3]. In early studies on enhancing drought tolerance in “San Deli”, our experimental group found that nitric oxide, a signaling substance, was effective [1, 3]. Following treatment with exogenous nitric oxide, “San Deli” was found to produce differential small RNAs in response to drought stress. It was discovered that MIR166 is particularly sensitive to both drought and NO [40]. MiR166 plays a crucial role in the formation [45–48]. It participates in the formation of Arabidopsis roots and maintains the abaxial fate of lateral organs by restricting the expression of HD-ZIP IIIs in the adaxial domain [45]. Likewise, the overexpression of MiR166a stimulated the growth of lateral roots in *Larix Leptolepis* seedlings [46]. In these transgenic roots, MIR166 overexpression resulted in a decrease in the number of symbiotic nodules and lateral roots, as well as the induction of ectopic development of vascular bundles [47]. Furthermore, transgenic Arabidopsis that overexpressed MIR166 displayed stunted growth and ultimately perished before producing rosette leaves. These plants lacked SAM and accumulated a significant amount of anthocyanin pigments. Alternatively, some plants exhibited mild growth retardation during the seedling stage but continued to grow into the reproductive phase [48]. MiR166 regulates both organogenesis and vascular development [47, 49, 50]. MiR166 is localized in the leaves, roots, and internode xylem of bamboo seedlings. It targets HOX and serves as a regulatory factor for the differentiation of bamboo vascular tissue [49]. MiR166 induced cambium differentiation in *Populus alba* by targeting HB1 under the regulation of the developmental process and season [50]. Furthermore, it has been discovered that mir166 plays a role in the production of secondary metabolites [51]. For instance, miR166 was found to be involved in the biosynthesis of piperine, a secondary metabolite in black pepper *(Piper nigrum L.)*, and the regulation of gallic catechin levels in tea trees [51]. MiR166 plays a crucial role in regulating plant growth, development, and responses to abiotic and biotic stresses. Research has shown that miR166 responds to drought stress in plants, including Arabidopsis [26]., Highbush Blueberry [29], and Soybean [30]. Furthermore, it was observed that STTM166 plants exhibited stronger drought resistance compared to wild-type plants [27, 28]. Additionally, some STTM166 plants displayed stunted growth while promoting lateral root development [26–28]. However, in high-bush blueberries, Vco-miR166s were upregulated under freezing, low temperature, and high temperature stress, affecting the plant’s response to abiotic stress [29]. In rice, MiR166 plays a crucial role in the accumulation and tolerance of Cd by regulating its target gene OsHB4 [35]. Regarding diseases, the sly-miR166/SlyHB module in tomatoes was found to be a negative regulatory factor for plant defense against Tomato leaf curl New Delhi virus (ToLCNDV) [52]. Under UV stress, MiR166 regulates auxin signaling by targeting auxin response factors (ARFs), which alters plant growth and development in response to UV stress [54]. However, there are few reports on the functional research of miR166 in alfalfa. Therefore, this study focuses on the bioinformatics analysis of MiR166 in alfalfa and predicts its target genes. The study analyzed the spatiotemporal expression of Msa-miR166s and their target genes in alfalfa roots and leaves under exogenous nitric oxide treatment in response to drought using qRT-PCR.

Previous studies have identified the miR166 family as an ancient class of miRNAs in plant evolution [25]. The distribution of members of the miR166 family in plants, as shown in Table 1, was analyzed statistically. Figure 2 illustrates that all branches exhibit clear conserved motifs, suggesting that Msa-miR166s are highly conserved. The evolutionary analysis of plant pre-miR166s is consistent with previous research [25, 27]. MiR166 is present throughout the plant kingdom, with no significant difference in distribution between dicotyledonous and monocotyledonous plants (see Table 1). However, MiR166 has the same number of mature bodies as its ancestors in older species, such as mosses, ferns, and gymnosperms. In angiosperms, the excess of mature bodies compared to the number of precursors suggests that some MiR166 precursors cleave into two distinct mature bodies that are retained [55]. It is suggested that the conservation of an increasing number of MiR166-5p mature bodies has contributed to the adaptation of angiosperm plants to their environment. The symptoms of leaf yellowing caused by potato virus X on Nicotiana benthamiana were reduced and virus accumulation decreased when nbe-miR166h-p5 was suppressed [53]. The analysis of plant pre-miR166s evolution (Fig. 3A) provides a further explanation that the 3′ end of pre-miR166 contains a highly conserved motif. The conserved motif of the 5′ end is relatively unstable, particularly in the 5′ end of the pink branch (Fig. 2), which includes Msa-miR166e. Figure 3 illustrates that Msa-miR166a and Msa-miR166c originate from the 3p end of their precursors, while Msa-miR166e-5p and Msa-miR166g-5p originate from the 5p end of their precursors. The predicted target genes for Msa-miR166s revealed that HD ZIP III, a typical target gene family of mir166s [30, 45], was targeted by Msa-miR166a and Msa-miR166c. However, Msa-miR166e-5p and Msa-miR166g-5p, which were FRI and SF1 genes respectively, did not belong to the HD ZIP gene family. Combined with Fig. 2, Msa-miR166e is unstable at the 5-terminal end and it is hypothesized that the resulting Msa-miR166e-5p matrices may differ from typical miR166s in terms of function or mechanism of action. The combination of weak and non-functional alleles of the *FRIGIDA (FRI)* and *FLOWERING LOCUS C (FLC)* genes can significantly reduce a plant’s lifetime water consumption by controlling its flowering time [58]. Overexpression of FRI in Arabidopsis and Citrus can enhance plant drought tolerance [64]. FRI upregulates the expression of *FLOWERING LOCUS C (FLC)*, which is the main determinant of natural changes in flowering time in Arabidopsis [58]. The dominant allele of FRI endows plants with the need for vernalization, thereby enabling them to asexually overwinter [59]. Therefore, it is hypothesized that the retained pre-miR166 mature 5′ region may have helped plants adapt to new environments or be more suitable for reproductive survival during the evolution of the species. Previous research has demonstrated that the 5′ sequence upstream of the pri-miR166 gene undergoes more evolutionary changes that affect the spatiotemporal expression of miR166, resulting in functional variability [56]. This statement avoids the previously mentioned point. Previous research has shown that Csi-miR166g-3p is downregulated during drought stress in tea plants and is involved in root tip development [31]. In highbush blueberries, Vco-miR166h-3p and Vco-miR166i-3p showed a rapid response 2 hours after freezing, while Vco-miR166d-5p, Vco-miR166f-5p, and Vco-miR166h-3p exhibited a strong response 12 hours after freezing [29]. Both MiR166–3p and -5p play a crucial role in plant stress response, although they exhibit differences in their timing and degree of stress response.

The secondary structure prediction of Msa-pre-miR166 (Fig. 3A) predicts that Msa-miR166c has the smallest free energy and is more likely to form folded precursor sequences than other Msa-miR166s. The variation in the number of stem-loops formed by each member of the miR166 family may be attributed to differences in their nucleotide sequences, which could impact the frequency of their activity [55]. The protomer has a longer free sequence and forms more unpaired regions, resulting in higher free energy [57], whereas miR166c has the lowest free energy and is thought to form folded precursor sequences relatively easily. Analyzed by comparison in the miRbase database (Table 2), it was found that both known and newly discovered miR166s in alfalfa can be found in other species with the same known mature miR166 base sequence. It can be seen that in the evolution of miR166s in the entire plant, the miR166 family of alfalfa is very conservative, and the miR166s with higher similarity are mostly from plants such as barrel medic, soybean, Maize, and Rice. According to current research on miR166s in these species [26, 27, 30], it has been found that plants respond to sudden drought stress by downregulating miR166s. The phylogenetic tree of mature miR166s in some species shows that Msa-miR166 has the closest genetic relationship with dicotyledonous leguminous plants such as Barrel Medic and Soybean, followed by monocotyledonous plants such as Maize and Rice in the gramineae family. This is consistent with the results of the analyses in Table 2. In the phylogenetic tree, Msa-miR166a and Msa-miR166d of the Msa-miR166 members seem to be more distantly related. There is evidence of evolutionary convergence between Msa-miR166a and Msa-miR166d during maturity, which may have contributed to alfalfa’s adaptation to its current environment (see Fig. 4). The mature miR166s phylogenetic tree exhibits a sequential evolutionary pattern. Msa-miR166s are found in different branches of the evolutionary tree, demonstrating the diversity of their evolution. Studies on miR166s in Maize [26], Highbush blueberries [29], and soybean [30], which are closely related to the evolution of Msa-miR166s, have shown that under drought conditions, the drought tolerance of plants is enhanced when the expression of MiR166s decreases.

To investigate the correlation between Msa-miR166s and drought, we selected six mature Msa-miR166s and examined their abundance in response to abiotic stress. Simultaneously, to confirm the accuracy of degradation sequencing, we selected six corresponding target genes and verified their relative expression with Msa-miR166s.

After 6 hours of drought treatment in alfalfa, there was a downregulation of miR166 expression, with varying degrees of decline. The data suggest that the expression of Msa-miR166s is downregulated upon the sudden onset of drought stress. This finding is consistent with previous research conducted on Maize [26], Highbush blueberries [29], and Soybeans [30]. The differences in exercise functions and methods may account for the varying degrees of downregulation among Msa-miRNA166s [57]. However, after 24 hours of drought, the expression of Msa-miR166s rebounded. Presumably for two reasons. The down-regulated expression of miR166s has been effective in coping with short-term drought [28,29,30,]. However, for the normal growth and development of alfalfa, the expression of miR166s must return to normal levels to cope with drought stress through other pathways [27, 32, 33]. Previous studies indicate that regulation between miRNA-target genes is dynamic, rather than exhibiting sustained high expression or silencing [58]. Once the target genes’ expression is activated, maintaining the baseline expression level of miR166s is sufficient to produce sustained regulation of the target genes.

Exogenous NO significantly reduced the expression of miR166s and enhanced their fluctuation, indicating that NO can effectively enhance drought tolerance in alfalfa by down-regulating miR166s. Additionally, it enhanced the response of Msa-miR166s to drought. These papers are in agreement with those of previous authors who found that NO promotes plant response to drought stress by regulating physiological and biochemical indices [4, 5, 40]. It is suggested that the application of exogenous NO may upregulate the expression of miR166s, potentially avoiding the adverse traits observed in STTM166 plants when miR166s are downregulated or silenced [26]. This could be beneficial for plant growth and development in normal environments [27]. Furthermore, similar to plants such as Highbush Blueberry [29], Msa-miR166s were expressed in both leaves and roots, indicating that Msa-miR166 plays a role in both organs during the seedling stage. Research has demonstrated that miR166 plays a crucial role in preserving the growth of plant vascular tissues, including the creation of secondary tissues like leaf vein development [26, 27] and root secondary growth [49, 50]. It is presumed that miR166 plays a similar role in alfalfa. Regarding the performance of STTM166 plants in Arabidopsis [26] and Maize [28] during drought, it is hypothesized that alfalfa may cope with short-term drought by regulating secondary growth rate and mobilizing resources throughout the plant body to ensure survival. In suitable living environments for alfalfa, external application of NO upregulates the expression of miR166s. However, under drought stress, Msa-miR166s are downregulated to cope with the stress. External application of NO significantly downregulates the expression of Msa-miR166s in the root.

Under normal conditions, the relative expression level of Msa-miR166s in roots and leaves is the same. However, after drought treatment, the expression level of Msa-miR166s in leaves fluctuates significantly, indicating that leaves are relatively more sensitive to drought. This suggests that the expression of Msa-miR166s in leaves and roots is spatiotemporally specific after drought treatment. At the same time, the relative expression levels of target genes showed opposite trends to Msa-miR166s.The accuracy of the degradation sequencing was verified, and it can be speculated that under the regulation of exogenous nitric oxide, the expression levels of Msa-miR166s decreased, reducing the cleavage of target gene mRNA and releasing enough target gene mRNA [54, 57], resulting in normal expression of target genes and enhancing drought resistance of alfalfa. Consistent with research in Tomatoes [33], Rice [35] and Arabidopsis [45], Degradome Sequencing showed that the Msa-miR166s mainly targets *the HD-ZIP-III gene family*, like *homeobox-leucine zipper protein REVOLUTA (REV)*, *homeobox-leucine zipper protein ATHB-8 (ATHB-8), homeobox-leucine zipper protein ATHB-15 (ATHB-15).* In addition, Msa-miR166s are generally targeted towards *the Pumpive o-succinyl benzoate-CoA ligase (CoA)*. It is hypothesized that *Ms-ATHB-8* and *Ms-REV* have similar functions in enhancing drought tolerance in alfalfa due to their similar predicted protein structures (Supplementary Table 5). It is speculated that the downregulation of mir166s expression mediated the upregulation of target gene *HD ZIP III*, which may help plants cope with drought stress by regulating processes such as vascular development, leaf polarity development, and root development. However, the expression level of target genes in the roots is a hundred times higher than that in the leaves. After drought treatment, it is in a continuous fluctuation state. This series of fluctuations may be due to the complete polarity development of the leaves [60, 61], while the development of the roots is still ongoing [62, 63], and a large number of *HD ZIP III* genes are still needed in the roots.

Furthermore, the target gene of miR166, as well as shear factors, may contribute to drought response through activities closely related to plant growth and development. For instance, Pumpive o succinylbenzoate CoA ligase is involved in the biosynthesis of secondary metabolites [65]. Splicing factor-like protein 1 (SF1) is a crucial protein factor required for the formation of spliceosomes, which regulate mRNA splicing. SF1 is also necessary during development in response to abscisic acid [66].

## Conclusion

In response to short-term drought, Msa-miR166s down-regulate expression in alfalfa (*Medicago sativa L.*). Exogenous nitric oxide can reduce the expression of Msa-miR166s in response to short-term drought. These findings suggest that Msa-miR166e-5p is responsive to environmental changes. The expression levels of target genes showed an opposite trend to Msa-miR166s, verifying the accuracy of Degradome sequencing in the early stage. This suggests that alfalfa experiences drought stress when regulated by exogenous nitric oxide, targeting *HD ZIP-III, FRI, and CoA ligase* genes. Additionally, the expression of Msa-miR166s in response to drought stress varies between leaves and roots, indicating spatiotemporal specificity.

### Supplementary Information

**Additional file 1: Figure S1-S2. Figure S1.** Picture of target gene degradation sites of Msa-miR166s (T-plots picture). **Figure S2**. Total cDNA prep gel images.

**Additional file 2: Table S1-S4. Table S1.** Precursor sequence of Msa-miR166s. **Table S2.** RT-qPCR primer sequences for Msa-miR166s. **Table S3.** The gene sequence targeted by Msa-miR166s. **Table S4.** RT-qPCR primer sequences for the target genes. **Table S5.** Secondary Structure of the Target Genes.

## Data Availability

The alfalfa genome of “Xinjiang Daye” varieties data used in this experiment were downloaded at (https://figshare.com/projects/whole_genome_sequencing_and_assembly_of_Medicago_sativa/66380), and the number of miR166s mature bodies and precursors in plants, the sequence of miR166s mature bodies in plants, and the precursor sequences of Arabidopsis and leguminous plants are all from the miRbase database (http://www.miRbase.org/). All data generated or analyzed during this study are included in this published article/Supplementary Material, further inquiries can be directed to the corresponding authors.
