# Supplementary figures and images for: Mechanism of action of microRNA166 on nitric oxide in alfalfa (Medicago sativa L.) under drought stress
Source: BMC Genomics. 2024 Mar 28;25:316. doi: 10.1186/s12864-024-10095-7 (PMC10976769; doi:10.1186/s12864-024-10095-7)

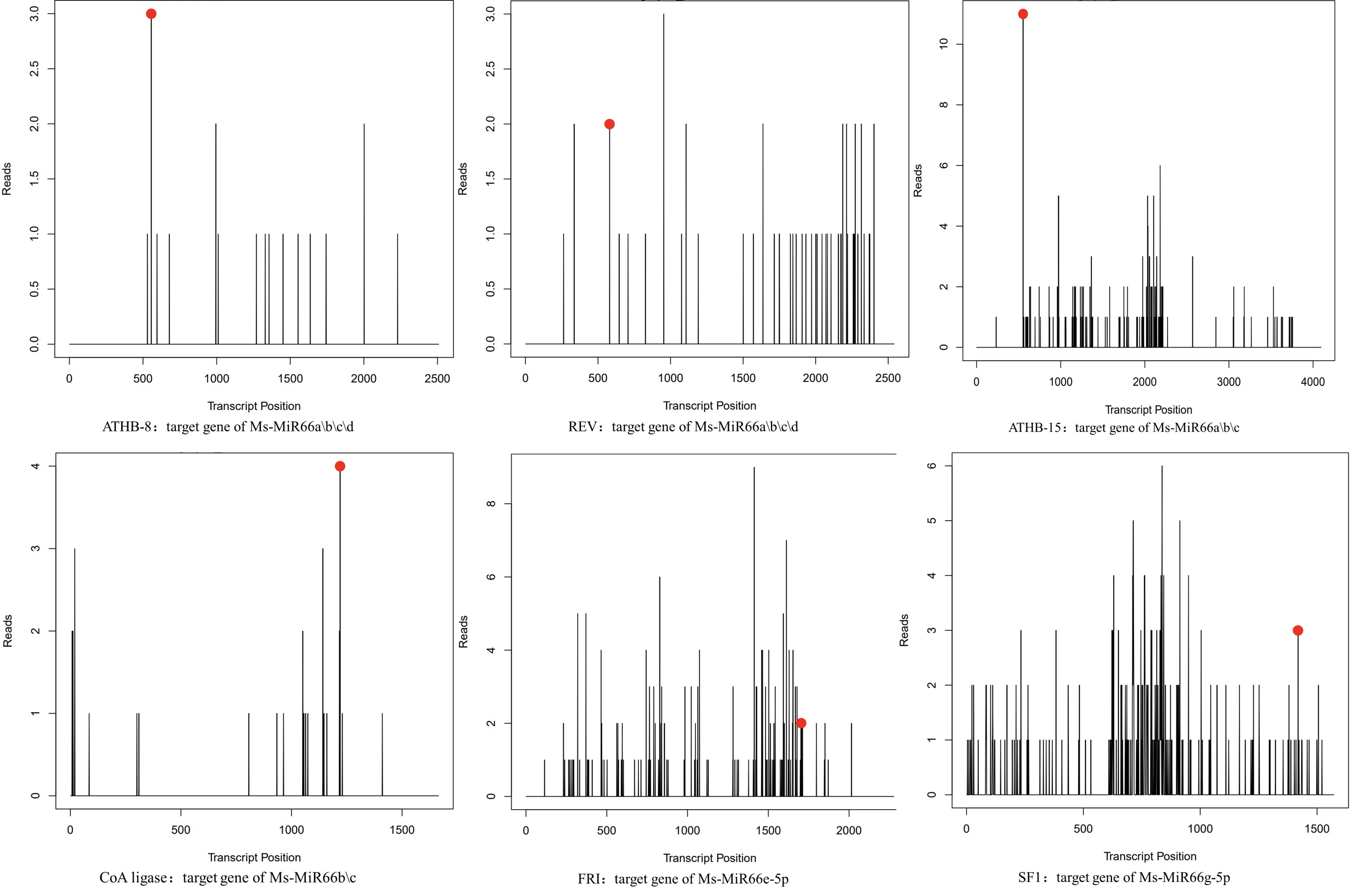

Supplement: Supplementary file 1 — Additional file 1: Figure S1-S2. Figure S1. Picture of target gene degradation sites of Msa-miR166s (T-plots picture). Figure S2. Total cDNA prep gel images. [file 12864_2024_10095_MOESM1_ESM.zip › supplementary figure/FigureS1. Picture of target gene degradation sites of Alfalfa the miR166s(T-plots picture).jpg]

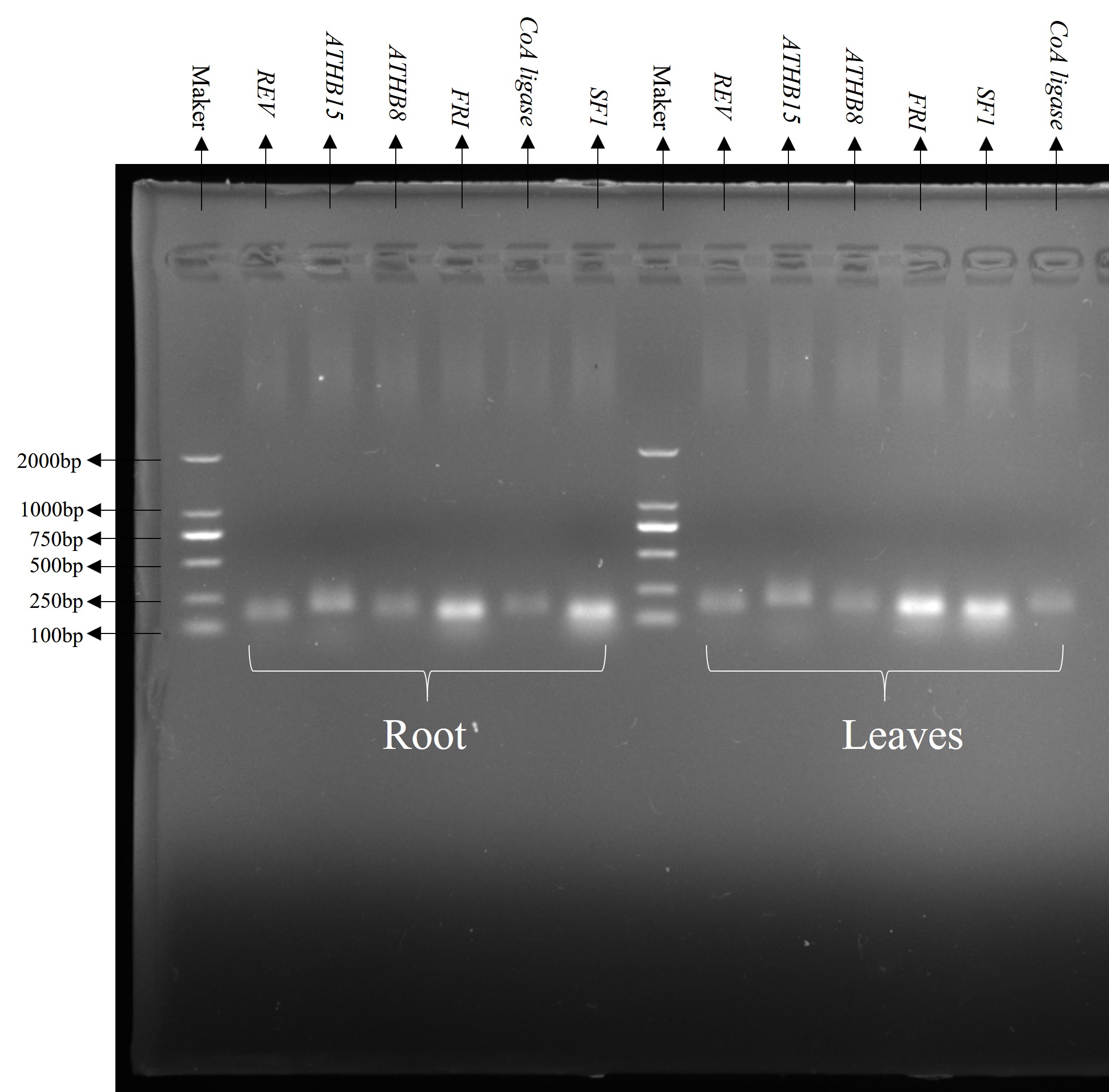

Supplement: Supplementary file 1 — Additional file 1: Figure S1-S2. Figure S1. Picture of target gene degradation sites of Msa-miR166s (T-plots picture). Figure S2. Total cDNA prep gel images. [file 12864_2024_10095_MOESM1_ESM.zip › supplementary figure/FigureS2. Total cDNA prep gel images.jpg]
